# Construction of an Overexpression Library for Chinese Cabbage Orphan Genes in *Arabidopsis* and Functional Analysis of *BOLTING RESISTANCE 4*-Mediated Flowering Delay

**DOI:** 10.3390/plants14131947

**Published:** 2025-06-25

**Authors:** Ruiqi Liao, Ruiqi Zhang, Xiaonan Li, Mingliang Jiang

**Affiliations:** 1 Molecular Biology of Vegetable Laboratory, College of Horticulture, Shenyang Agricultural University, Shenyang 110866, China; lrq1602144472@163.com (R.L.); rqqq3893@163.com (R.Z.); 2 School of Agriculture, Jilin Agricultural Science and Technology College, Jilin 132101, China

**Keywords:** Chinese cabbage, orphan genes, *Arabidopsis*, overexpression library, delayed flowering

## Abstract

Orphan genes (*OGs*), which are unique to a specific taxon and have no detectable sequence homology to any known genes across other species, play a pivotal role in governing species-specific phenotypic traits and adaptive evolution. In this study, 20 *OGs* of Chinese cabbage (*Brassica rapa OGs*, *BrOGs*) were transferred into *Arabidopsis thaliana* by genetic transformation to construct an overexpression library in which 50% of the transgenic lines had a delayed flowering phenotype, 15% had an early flowering phenotype, and 35% showed no difference in flowering time compared to control plants. There were many other phenotypes attached to these transgenic lines, such as leaf color, number of rosette leaves, and silique length. To understand the impact of *BrOGs* on delayed flowering, *BrOG142OE*, which showed the most significantly delayed flowering phenotype, was chosen for further analysis, and *BrOG142* was renamed *BOLTING RESISTANCE 4* (*BR4*). In *BR4OE*, the expression of key flowering genes, including *AtFT* and *AtSOC1*, significantly decreased, and *AtFLC* and *AtFRI* expression increased. GUS staining revealed *BR4* promoter activity mainly in the roots, flower buds and leaves. qRT-PCR showed that *BR4* primarily functions in the flowers, flower buds, and leaves of Chinese cabbage. BR4 is a protein localized in the nucleus, cytoplasm, and cell membrane. The accelerated flowering time phenotype of *BR4OE* was observed under gibberellin and vernalization treatments, indicating that *BR4* regulates flowering time in response to these treatments. These results provide a foundation for elucidating the mechanism by which *OGs* regulate delayed flowering and have significance for the further screening of bolting-resistant Chinese cabbage varieties.

## 1. Introduction

Orphan genes (*OGs*) refer to genes found in a species that have no sequence similarity to genes in a particular database or to other known open reading frames [1,2]. *OGs* exist widely in all areas of life, including in microbes [3,4], plants [5,6], primates [7] and insects [8]. Although most *OGs* have unknown functions, and often lack identifiable functional domains, they are relevant in the fields of genomics, genetics, comparative biology, structural biology, phylogenetic biology, and evolution. However, it is particularly difficult to study *OGs* using traditional methods. Nonetheless, there is sufficient evidence to show that *OGs* have numerous functions [4,9,10]. Their expression is highly specific and at a low level, *OGs* participate in a variety of biological and abiotic stress responses, regulating substance metabolism and affecting species-specific evolution [11,12,13]. The first *Arabidopsis OG* identified in plants, *Qua-Quine Starch* (*AtQQS*, *AT3G30720*), regulates the distribution of carbon and nitrogen in *Arabidopsis* leaves. The starch content in its RNA interference lines significantly increased, but the protein content significantly decreased [14,15,16,17]. Some *OGs* improve plant adaptability to abiotic stress. Transgenic *Arabidopsis* heterologously expressing *OG ABA-RESPONSIVE DROUGHT TOLERANCE* (*PpARDT)* exhibits improved drought tolerance and is more responsive to exogenous abscisic acid (ABA) than the wild type [18]. The interaction between FUSARIUM RESISTANCE ORPHAN GENE (TaFROG) and SUCROSE NON-FERMENTING1-RELATED KINASE1 (TaSnRK1) improves resistance to *Fusarium graminearum* in wheat (*Triticum aestivum*) [19]. *OG BOLTING RESISTANCE 1* (*BR1*), which has been identified in Chinese cabbage, is a flowering time regulation gene that can improve the bolting resistance of plants [20]. These characteristics demonstrate that *OGs* perform essential regulatory functions in plant life activities and improve plant stress resistance and quality by affecting specific traits. However, the function of *OGs* in Chinese cabbage needs to be studied further.

Flowering time is an important agronomic trait affecting reproduction and product quality. Internal physiological factors and external environmental factors jointly affect some key flowering traits and regulate flowering time [21]. In *Arabidopsis*, six flowering pathways control the flowering stage, namely, the photoperiod, vernalization, autonomous, gibberellin (GA), age, and temperature pathways, which are both independent and related to each other and jointly act on several specific key genes [22,23,24,25,26]. *FLOWERING LOCUS C* (*FLC*), encoding a MADS-box transcription factor, acts as a flowering repressor whose expression is suppressed by vernalization, thereby promoting flowering in plants. *FRIGIDA* (*FRI*) enhances the inhibition of *FLC* [27,28]. In the GA signaling pathway, FLOWER LOCUS T (FT) interacts with bZIP family transcription factor FLOWER LOCUS D (FD) to activate downstream flower meristem recognition genes, including *SUPPRESSOR OF OVEREXPRESSION OF CONSTANS 1* (*SOC1*), *LEAFY* (*LFY*), *and APETALA 1* (*AP1*), which are expressed in the plant stem apex meristem and affect flowering [29,30]. Although these findings establish a fundamental framework for the study of flowering time in plants, the relationship between the pathways controlling flowering time is not yet clear, and there are few studies indicating how *OGs* regulate flowering time.

Chinese cabbage is a crop with high economic and nutritional value that is widely cultivated in China. Premature bolting significantly compromises Chinese cabbage product quality, necessitating comprehensive investigation of the molecular mechanisms underlying bolting resistance regulation in this economically important crop. *SET DOMAIN GROUP 8* (*BrSDG8*) in Chinese cabbage is a homologous gene of *AtSDG8* in *Arabidopsis* that encodes a protein affecting H3K4me3 in *FLC* chromatin. An allelic mutant verified that *BrSDG8* mutation leads to early bolting [31]. The histone H4 protein BrHIS4 was screened from a yeast two-hybrid screening library using *VERNALISATION INSENTIVE 3.1* (*BrVIN3.1*) as bait. *BrHIS4* overexpression led to premature flowering in Chinese cabbage under normal growth conditions, and the expression of photoperiod-related flowering genes was reduced through the ABA signaling pathway under drought conditions, preventing premature bolting [32]. *AtFLC* controls the flowering time of *Arabidopsis*, and *FLC* has been found in Chinese cabbage with multiple copies: *BrFLC1* on chromosome A10, *BrFLC3* and *BrFLC5* on chromosome A03, and *BrFLC2* on chromosome A02. BrSOC1b likely regulates flowering by interacting with multiple MADS-box proteins, including AGAMOUS-LIKE 9a (BrAGL9a), BrAGL9b, BrAGL2, and BrAGL8 proteins [33,34]. Although the roles of *BrOGs*, such as *BR1* [20], *BOLTING RESISTANCE 2* (*BR2*) [35], and *BOLTING RESISTANCE 3* (*BR3*) [15], in bolting and flowering have been revealed [36], further research is needed to screen and identify *BrOGs* that regulate bolting resistance in Chinese cabbage and to determine their specific mechanisms of action.

In this study, 20 *OGs* from Chinese cabbage were transferred into *Arabidopsis* through genetic transformation to construct an overexpression library. After conducting a detailed phenotypic investigation of these *BrOGsOE* lines, *BrOG142OE*, which showed the most delayed flowering phenotype, was selected for further study, and *BrOG142* was renamed *BOLTING RESISTANCE 4* (*BR4*). The expression pattern and subcellular localization of *BR4* were determined. The flowering time and related characteristics of the *BR4OE* lines were analyzed under different treatments. In addition, the expression patterns of key flowering genes were analyzed using quantitative real-time polymerase chain reaction (qRT-PCR). This study evaluated the specific pathways by which *BR4* regulates flowering, providing new insights into the function of *OGs* and a new approach to breeding bolting-resistant varieties of Chinese cabbage.

## 2. Results

### 2.1. Construction and Phenotypic Investigation of a BrOGs Overexpression Library in Arabidopsis

Our previous study screened and identified *BrOGs* in Chinese cabbage [37]. We randomly selected 20 *OGs* for bioinformatics analysis, among which two were located on chromosome A10 and eighteen were located on scaffolds, and the number of exons ranged from one to five (Table S1). Therefore, to study the function of these *OGs*, the 20 *OGs* from Chinese cabbage ‘Chiifu’ were amplified, and overexpression vectors were constructed. The recombinant plasmid was transferred into *Agrobacterium tumefaciens* GV3101 (Figure S1) for genetic transformation of wild-type *Arabidopsis* (WT) using the floral dip method. The T_3_ transgenic *BrOGsOE* lines were obtained using successive multi-generational self-crossing.

**Figure 1:**
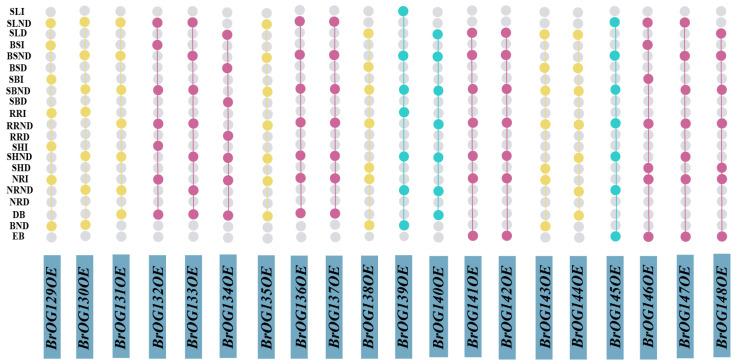
Summary figure of the phenotypic investigation of *Arabidopsis* transgenic plants. The blue bar chart in the figure represents the aggregate results of the multiple phenotypes of different transgenic lines. Purple circles represent lines that bloom late and have other phenotypes. Blue circles represent lines that bloom earlier and have other phenotypes. Yellow circles represent lines with no difference in flowering time but with other phenotypes. Gray circles represent transgenic plants that do not have the corresponding phenotype. EB represents early bolting; BND represents no difference in bolting; DB represents delayed bolting; NRD represents a decreased number of rosette leaves; NRND represents no difference in the number of rosette leaves; NRI represents an increased number of rosette leaves; SHD represents a decrease in stem height; SHND represents no difference in stem height; SHI represents an increase in stem height; RRD represents a decrease in the rosette radius; RRND represents no difference in the rosette radius; RRI represents an increase in the rosette radius; SBD represents a decrease in the number of main stem branches; SBND represents no difference in the number of main stem branches; SBI represents an increase in the number of main stem branches; BSD represents a decrease in the number of main branch siliques; BSND represents no difference in the number of main branch siliques. BSI represents the increase in the number of main branch siliques; SLD represents a decrease in the silique length; SLND represents no difference in the silique length; SLI represents an increase in the silique length. These phenotypes were in comparison to WT.

**Figure 2:**
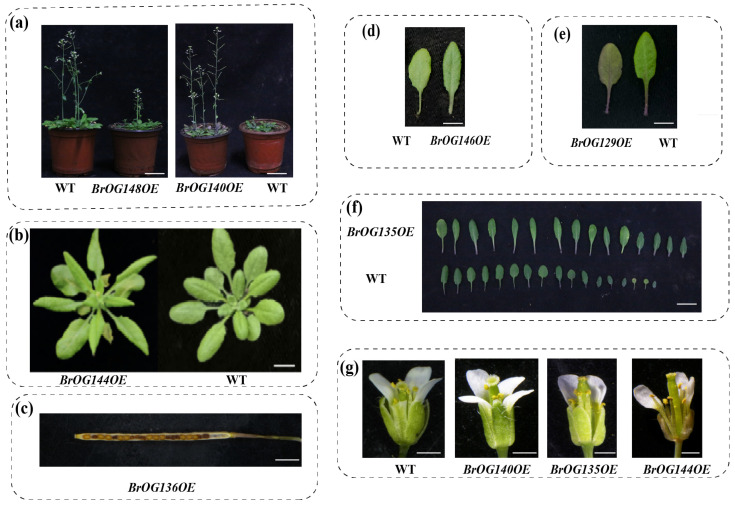
Phenotypes of transgenic *Arabidopsis* plants. (**a**) Flowering stage comparison among *BrOG148OE*, *BrOG140OE*, and WT (bar = 10 mm). (**b**) Phenotype comparison between *BrOG144OE* and WT (bar = 10 mm). (**c**) Mature silique of *BrOG136OE* (bar = 1 mm). (**d**) Leaf color comparison between *BrOG146OE* and WT (bar = 10 mm). (**e**) Leaf color comparison between *BrOG129OE* and WT (bar = 10 mm). (**f**) Leaf shape comparison between *BrOG135OE* and WT (bar = 10 mm). (**g**) Floral organ comparison among *BrOG140OE*, *BrOG135OE*, *BrOG144OE*, and WT (bar = 1 mm).

Vegetative growth and reproductive growth traits were investigated in *Arabidopsis BrOGsOE* transgenic lines. Among them, the delayed flowering phenotype was abundant, accounting for about 50% of the lines, and the early flowering phenotype accounted for 15% (Figure 1). Approximately 35% of transgenic plants exhibited no difference in flowering time compared to WT. In addition to flowering time, many other phenotypes were attached to these transgenic lines (Figure 2). For example, compared to WT, *BrOG144OE* showed no difference in flowering time, but its stem tips and floral organs were yellow, the female and male differed in length, and the leaves were shriveled (Figure 2b,g). *BrOG136OE* had delayed flowering and poor fertility (Figure 2c). *BrOG146OE* exhibited delayed flowering, an increased number of rosette leaves at flowering, and greener leaves (Figure 2d). *BrOG129OE* showed no difference in flowering time, but more anthocyanins accumulated in its leaves (Figure 2e). Thus, *BrOGs* play multifaceted roles in diverse physiological processes during plant growth and development.

### 2.2. Phenotypic Identification of BR4OE Lines

**Figure 3:**
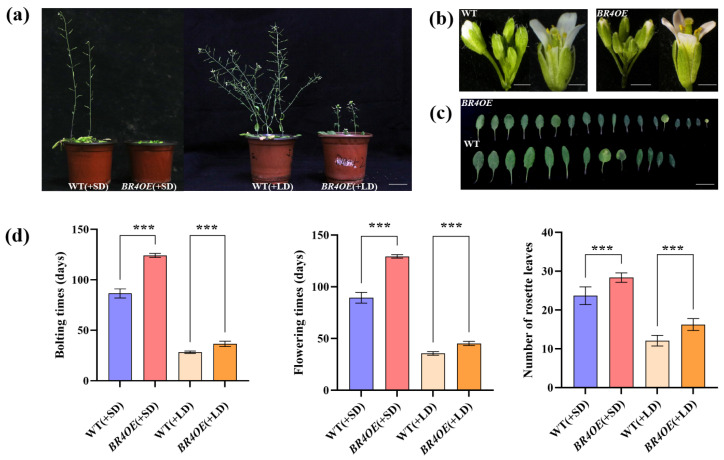
Phenotypic characterization of *BR4OE* lines. (**a**) Phenotypes of WT and *BR4OE* plants under short day (SD) (left)/long day (LD) conditions (right) (bar = 10 mm). (**b**) Floral organ phenotypes of WT and *BR4OE* (bar = 1 mm). (**c**) Leaf phenotypes of WT and *BR4OE* (Bar = 10 mm). (**d**) Bolting time, flowering time, and rosette leaf number of WT and *BR4OE* under SD/LD conditions. Experiments were repeated three times (*n* ≥ 10), and asterisks (***) indicate a significant difference (*p* < 0.001) from WT, as shown by a Student’s *t*-test.

During Chinese cabbage breeding, delayed flowering time can improve leafy head quality and yield. *BrOG142OE* lines showed the most delayed flowering time in this overexpression library (Figure S2), and *BrOG142* was renamed *BOLTING RESISTANCE 4* (*BR4*) and selected for further analysis. Compared to WT, *BR4OE* exhibited small leaves and floral organs, significantly delayed bolting and flowering times, and produced more rosette leaves when flowering (Figure 3). The mechanism by which *BR4* regulates bolting resistance requires further analysis.

### 2.3. Expression Analysis of Key Flowering Genes

**Figure 4:**
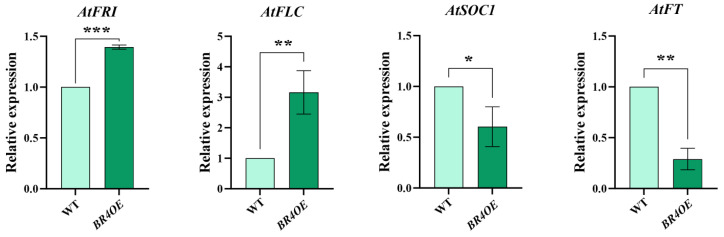
Expression of flowering-related genes. All data are shown as the mean ± SD of three biological replicates. Asterisks indicate a significant difference from WT, as shown by a Student’s *t*-test, * *p* < 0.05, ** *p* < 0.01, *** *p* < 0.001.

*BR4* overexpression in *Arabidopsis* delayed flowering; qRT-PCR was used to further analyze the transcription levels of key flowering genes. Compared to WT, the expression of *AtFLC* and *AtFRI* genes in *BR4OE* significantly increased, while the expression of *AtSOC1* and *AtFT* significantly decreased (Figure 4). The delayed flowering phenotype of *BR4OE* lines correlates with altered expression of flowering-related genes, implying a possible role for *BR4* in modulating flowering time.

### 2.4. BR4 Gene Sequence Analysis

*BR4* was obtained from the Chinese cabbage cultivar ‘Chiifu’. The total length of *BR4* was 207 bp, and *BR4* was an intron-less gene. The protein prediction results showed that BR4 had a molecular weight of 7157.08, a theoretical isoelectric point of 5.97, an amino acid residue base of 68, and an instability coefficient of 69.74. The predicted protein structure showed four types of structures: α-helix (5.88%), extended chain (19.12%), β-corner (8.82%), and random curl (66.18%). Sequence analysis of the BR4 protein showed no conserved domains. These results indicate that *BR4* represents a previously uncharacterized gene with an unknown function.

### 2.5. BR4 Promoter-Driven GUS Expression and Its Regulatory Patterns During the Flowering Stage

**Figure 5:**
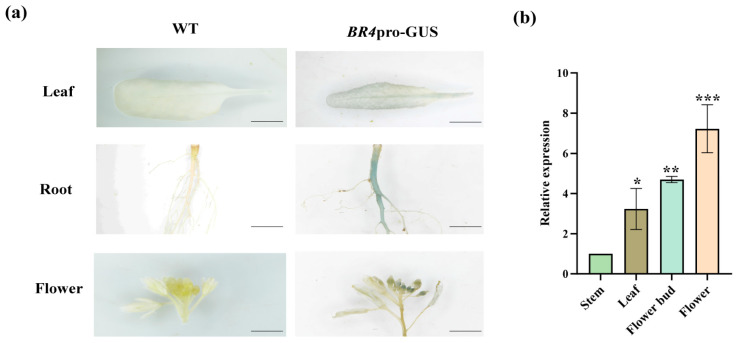
Analysis of the expression characteristics of *BR4*. (**a**) GUS staining of the *BR4* promoter (bar = 1 mm). (**b**) Expression levels of *BR4* in various tissues during the flowering stage. All data are shown as the mean ± SD of three biological replicates. Asterisks indicate a significant difference from WT, as shown by a Student’s *t*-test, * *p* < 0.05, ** *p* < 0.01, *** *p* < 0.001.

The constructed 1301-*BR4*pro-GUS fusion expression vector was transferred to *Arabidopsis*. Positive plants were screened in 1/2 Murashige and Skoog (MS) medium containing hygromycin and verified through PCR amplification (Figure S3). GUS staining showed that blue signals were detected in the roots, flowers, and leaves of transgenic *Arabidopsis* lines, and stem tissues showed no detectable GUS activity (Figure 5a). During the flowering stage, *BR4* expression in the Chinese cabbage ‘GT-24’ was analyzed using qRT-PCR. *BR4* had the highest expression level in the flowers, followed by the flower buds and leaves, and the lowest expression level was detected in the stem (Figure 5b). These findings suggest that *BR4* may participate in flowering stage progression and flowering time regulation.

### 2.6. Subcellular Localization of the BR4 Protein

**Figure 6:**
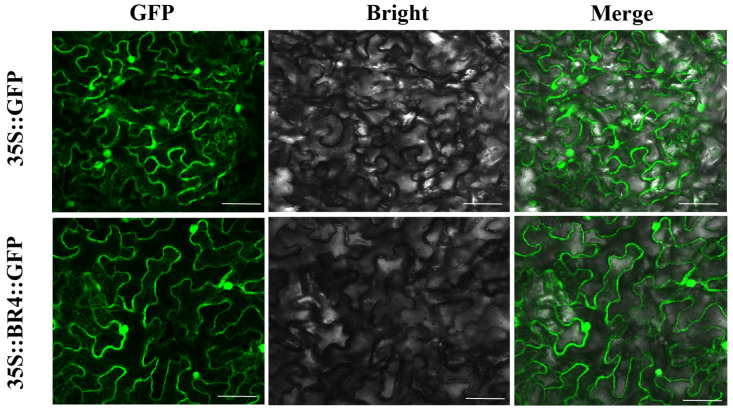
Localization of the BR4 protein in tobacco leaf cells. A Leica confocal microscope was used to collect images 48 h after agro-infiltration. Bar = 75 μm.

Fusion expression vectors 35S::BR4::GFP and 35S::GFP driven by the CaMV35S promoter were used for subcellular localization analysis. Green fluorescence signals were detected in the cytoplasm, cell membrane, and nucleus of BR4-GFP (Figure 6); thus, the BR4 protein localized to the cytoplasm, nucleus, and cell membrane.

### 2.7. BR4 Regulates Flowering Time in Response to GA and Vernalization Treatments

**Figure 7:**
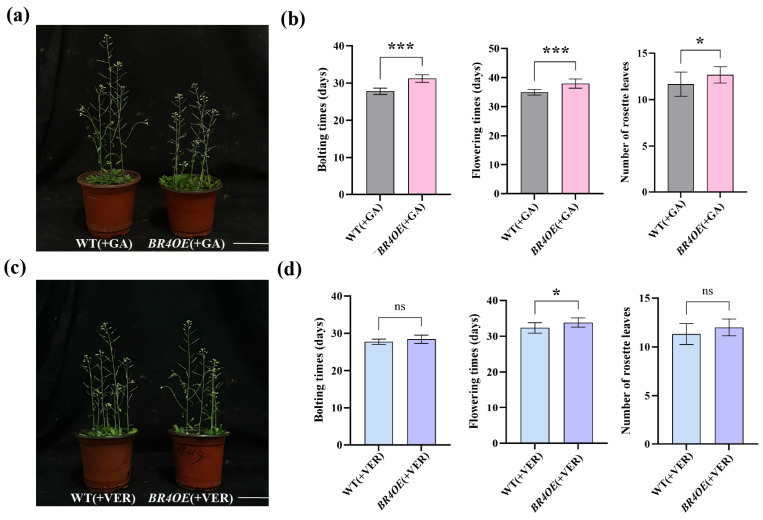
Flowering time of *BR4OE* and WT plants under different treatments. (**a**,**b**) Phenotypic observation of WT and *BR4OE* under GA_3_ treatment. (**c**,**d**) Phenotypic observation of WT and *BR4OE* under vernalization treatment. All data are shown as the mean ± SD of three biological replicates. Asterisks indicate a significant difference from WT, as shown by a Student’s *t*-test; ns indicates no difference, ^ns^ *p* > 0.05, * *p* < 0.05, *** *p* < 0.001. Ver represents vernalization treatment.

*BR4OE* and WT plants were treated with GA_3_ and vernalization, and the bolting time, flowering time, and number of rosette leaves were investigated. Compared to WT, GA_3_ treatment accelerated the bolting and flowering times of *BR4OE* (Figure 7a,b). After vernalization treatment, the bolting time, flowering time, and number of rosette leaves in *BR4OE* lines almost returned to levels observed in WT plants (Figure 7d). Therefore, *BR4* may regulate flowering time by responding to both GA and vernalization treatments.

### 2.8. BR4 Expression Level Under Exogenous Treatment

**Figure 8:**
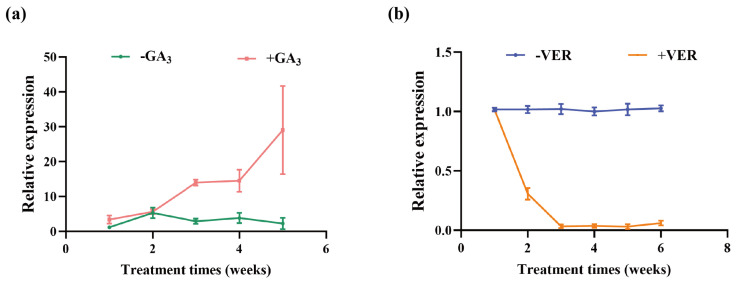
*BR4* expression levels in Chinese cabbage under different treatments: (**a**) GA_3_ treatment, (**b**) vernalization treatment. Ver represents vernalization treatment.

The expression level of *BR4* under GA_3_ and vernalization treatments was analyzed. In contrast to the control group, *BR4* expression gradually increased with an increase in GA_3_ spraying times (Figure 8a). Meanwhile, under vernalization treatment, *BR4* expression showed a significant downward trend with the extension of low-temperature treatment time (Figure 8b). Therefore, *BR4* expression is regulated by both GA_3_ and vernalization treatments, suggesting its involvement in flowering time regulation through GA and vernalization pathways.

## 3. Discussion

Flowering time is closely related to the biological yield and seed quality of Chinese cabbage [38,39]. There are few studies on *OGs*, and no functional domains can be identified, making it difficult to characterize their functions. It is particularly difficult to study *OGs* using traditional methods, but gene sequences can be obtained using reverse genetics, which can result in the rapid annotation of gene functions [40]. Although *Arabidopsis* and Chinese cabbage belong to the Brassicaceae family, comparative genomic analysis has revealed that Chinese cabbage *OGs* lack detectable sequence homology with any *Arabidopsis* genes; thus, the *Arabidopsis* flower dipping method is more efficient than traditional methods [41]. In this study, 20 Chinese cabbage *OGs* were randomly selected from previous studies to establish transgenic libraries in *Arabidopsis* and to explore the functions of *OGs* in regulating flowering time. Of the transgenic strains, 50% showed delayed flowering. In our previous *BrOGs* overexpression library, 72.66% of transgenic *BrOGsOE* lines showed significant phenotypic changes [36], providing strong support for the findings of this study. Our results not only advance the functional characterization of *OGs* but also enable marker-assisted selection for targeted genetic improvement in Chinese cabbage breeding programs.

In this study, a new *OG, BR4,* was shown to positively regulate the flowering time of plants, further confirming the relationship between its expression specificity and flowering time. Sequence analysis showed that *BR4* is an unknown gene localized in the cell membrane, nucleus, and cytoplasm that regulates the expression of flowering traits. Similarly, *BR1* overexpression in *Arabidopsis* decreased the expression levels of flowering-related factors *AtSOC1*, *AtLFY*, and *FRUITFULL* (*AtFUL*) [20]. In addition, *BR2* is a cell membrane-localized protein that affects the expression of flowering-related genes *BrFRI*, *BrSOC1s*, *BrLFYs*, and *BrFTs* through the vernalization pathway and, thus, affects flowering time [35]. These studies support the findings of the present study. The changes in the flowering time of these transgenic plants may be related to the direct or indirect participation of these *OGs* in the flowering regulatory network, indicating that *OGs* play an indispensable role in plant flowering time, and its specific mechanism is worthy of further exploration.

Current research indicates that GA, vernalization, photoperiod, age, temperature, and autonomic pathways regulate flowering time [30,42,43,44]. Different genes have been found to regulate the flowering time of plants by responding to different flowering pathways. Our findings demonstrate that *BR4* modulates flowering time through its responsiveness to both GA and vernalization pathways. Treatment with exogenous GA_3_ leads to early flowering, as GA is the main regulator of these processes, acting through the GA signaling pathway [45]. The DELLA family gene *RGA-LIKE1* (*BraRGL1*) is the key regulator of flowering time in *B*. *rapa*. Exogenous GA_3_ treatment enhanced *BraSOC1* activation, suggesting that BraRGL1–BraSOC1 modulates bolting and flowering by controlling the expression of xyloglucan endotransferase (*BraXTH3*) and *BraLFY* [46]. Transcriptome analysis showed that *BcSOC1* expression is closely related to bolting in flowering Chinese cabbage, and exogenous GA_3_ and low-temperature treatment significantly upregulates *BcSOC1*, promoting bolting and flowering [47]. *FLC* is the core gene of the vernalization pathway in *Arabidopsis*. With the increase in the duration of cold temperature exposure, the expression of *FLC* mRNA gradually decreased, and histone H3K27me3 and H3K9me2 quantitatively accumulated in the *FLC* locus. *FLC* expression remains elevated in plants requiring vernalization, where stable epigenetic silencing is necessary to release floral repression and promote flowering [48]. In this study, compared to WT, the expression levels of the *AtFL*C and *AtFRI* genes in *BR4OE* were significantly increased, and the flowering time of *BR4OE* after vernalization was advanced. This evidence supports the findings of the present study, but the specific mechanism by which *BR4* regulates Chinese cabbage’s resistance to bolting still requires further investigation.

In this study, the roles of 20 *BrOGs* in regulating various traits were preliminarily identified, and the regulatory effects of *BR4* on the bolting resistance of Chinese cabbage were verified. However, the mechanism and functional characteristics of *BrOGs* require further study. Future studies should employ both overexpression and knockout genetic transformations to systematically validate the biological roles of these *BrOGs* in Chinese cabbage. This study provides new gene sources and references for identifying bolting-resistance genes and cultivating varieties of Chinese cabbage that are resistant to bolting.

## 4. Materials and Methods

### 4.1. Plant Materials and Cultivation Conditions

Detailed cultivation conditions for wild-type *Arabidopsis* (Col-0), Chinese cabbage inbred line ‘GT-24’, Chinese cabbage cultivar ‘Chiifu’, and tobacco (*Nicotiana benthamiana*) have been described in previous studies [49].

### 4.2. BrOGs Overexpression in Arabidopsis

Full-length *BrOGs* were amplified from Chinese cabbage ‘Chiifu’ and inserted into the *EcoR* I and *Xho* I restriction sites of the pBinGlyRed3-35S vector. The recombinant vector pBinGlyRed3-35s-BrOGs contained a DsRed marker gene (Discosoma red fluorescent protein), which was used to screen transgenic seeds with green fluorescence and a red filter. The recombinant vector was introduced into *Agrobacterium tumefaciens* GV3101 using the freeze–thaw method, and *Arabidopsis* Col-0 was genetically modified using the flower dipping method [48] to obtain transgenic *BrOGsOE* lines. The primer pairs are shown in Table S2.

### 4.3. Phenotypic Investigation of BrOGsOE-Overexpressing Plants

Transgenic *BrOGsOE* plants were self-crossed for multiple generations to obtain T_3_ generation plants. Three biological replicates were used, and at least 10 plants were observed in each replicate. Bolting time was determined as the time from seeding to bud visibility, and flowering time was the time from seeding to the opening of the first flower. The number of rosette leaves of each plant was counted. The rosette radius was measured by taking the average length of the two largest fully expanded leaves of each plant. The number of siliques on the main branches and the length of the silique were measured using a previously described method [42]. The main stem branch number and stem height were measured after most of the seeds matured. Detailed methods refer to a previous study [37], and the phenotypes of floral organs were observed using a dissecting microscope (Nikon SMZ800, Tokyo, Japan).

### 4.4. Analysis of BR4 Sequence

The *BR4* gene sequence was analyzed using the *Brassica* database (BRAD, http://brassicadb.cn/). ProParam (https://web.expasy.org/protparam/ (accessed on 1 June 2024)) was used for amino acid analysis of physical and chemical properties. The protein secondary structures were predicted using the SOPMA database (http://npsa-pbil.ibcp.fr/cgi-bin/npsa_automat.pl?page=npsa_sopma_html (accessed on 3 June 2024)). The Conserved Domain Database (https://www.ncbi.nlm.nih.gov/) was used to compare conserved domains.

### 4.5. GUS Fusion Expression of the BR4 Promoter

The 2000-bp promoter sequence of the *BR4* gene was obtained from Chinese cabbage ‘Chiifu’. The promoter of the *BR4* gene was amplified, and the target fragment was purified and recovered (Tiangen, DP210831, Beijing, China). Plasmid pCAMBIA1301 was cut into linearized vectors with *Kpn* I and *Nco* I, and the fusion expression vector 1301-*BR4*pro-GUS was constructed. After being transformed into *Arabidopsis* using the floral dip method, the harvested seeds were sown on 1/2 MS medium containing hygromycin, and the transgenic plants were screened and identified using PCR. DNA extraction and PCR amplification were performed according to established protocols [37]. The plants were stained with a GUS staining kit (Coolaber, SL7160, Beijing, China) and observed under an optical microscope (Nikon, ECLIPSE 80i, Tokyo, Japan). The primer pairs are shown in Table S2.

### 4.6. Subcellular Localization Assays of BR4 Protein

The total RNA of ‘Chiifu’ leaves was extracted using the Trizol method and then reverse transcribed into cDNA. The plasmid 1302-GFP was linearized with *Kpn* I, and the fusion expression vector 1302-BR4-GFP was constructed. The recombinant fusion vector and empty vector were introduced into tobacco leaves via *A*. *tumefaciens*-mediated transient transformation. The constructed fusion expression vector and empty vector were injected into tobacco leaves using *A*. *tumefaciens* infiltration. The treated tobacco leaves were placed on a glass slide, and fluorescence signals were observed under a laser-scanning microscope (Leica Microsystems, Wetzlar, Germany). The excitation light of the green fluorescent protein (GFP) was set at 470 nm (primer pairs are shown in Table S2).

### 4.7. GA_3_ and Vernalization Treatments of Arabidopsis WT and BR4OE

For GA_3_ treatment, *Arabidopsis* WT and *BR4OE* were sprayed with 20 μmol/L GA_3_ 3 times per week until blossoming. In the vernalization treatment, germinated WT and *BR4OE* were grown at 4 °C for 30 d. At the same developmental stage, the phenotypes of WT and *BR4OE* plants were compared using previous methods [20]. At least 30 plants were used for each experiment.

### 4.8. GA_3_ and Vernalization Treatment of Chinese Cabbage

One-week-old Chinese cabbage ‘GT-24’ was sprayed with 500 mg/L GA_3_, and samples were collected 12 h after spraying, with a total of five applications. As a control, ‘GT-24’ was treated with an equal volume of distilled water. Three-week-old Chinese cabbage ‘GT-24’ was cultured at 4 °C. ‘GT-24’ under non-vernalization conditions was used as the control, and samples were collected once per week for six weeks. qRT-PCR was used to determine the expression level of *BR4* in ‘GT-24’, with three biological replicates and three technical replicates.

### 4.9. qRT-PCR Analysis

RNA extractions, cDNA synthesis, and qRT-PCR analysis were performed as described previously [50,51]. During the flowering stage, the total RNA of the stems, leaves, flowers, and flower buds of Chinese cabbage ‘GT-24’ and the leaves of *Arabidopsis* were extracted, and a reverse transcription experiment was performed using a reverse transcription kit (Takara, 6110A, Beijing, China). Primers were designed using Primer Premier v5.0 (Table S3). *Br18SrRNA* and *AtActin* were used as reference genes for qRT-PCR in Chinese cabbage and *Arabidopsis*, respectively. qRT-PCR experiments were performed using a Super Real PreMix Plus Kit (Tiangen, FP230630), with three biological replicates and three technical replicates, and qRT-PCR was performed using the QuantStudioTM 6 Flex System (ABI, Los Angeles, CA, USA).

### 4.10. Statistical Analysis

SPSS v19.0 software was used to compare data through Student’s *t*-tests. All data are shown as the mean ± SD of three biological replicates. Graphs were generated using GraphPad Prism software (v9.2).

## 5. Conclusions

An overexpression library was established in *Arabidopsis* using 20 *BrOGs*, laying a foundation for screening genes conducive to the molecular improvement of Chinese cabbage. The *BR4* gene has a potential role in regulating the flowering time of Chinese cabbage. *BR4OE* exhibited significant phenotypic alterations, including delayed flowering initiation and modified expression patterns of key flowering regulators compared to WT controls. At the same time, exogenous analysis revealed that *OG BR4* may be a new regulator of flowering time through the GA and vernalization pathways. These findings offer novel insights into the regulatory relationship between *OGs* and flowering time control in Chinese cabbage, thereby laying the theoretical groundwork for future research.

## Data Availability

All data supporting the findings of this study are available within the paper and within its Supplementary Materials published online.

## Supplementary Materials

The following supporting information can be downloaded at: https://www.mdpi.com/article/10.3390/plants14131947/s1, Figure S1. PCR detection results of *BrOG* cloning. Figure S2. Flowering time of *BrOGs* overexpression lines. Figure S3. Identification of GUS-positive plants. Table S1. Information on the 20 *BrOGs*. Table S2. Primer pairs used for vector construction in this study. Table S3. Primer pairs used for qRT-PCR in this study.

## Abbreviations

*AP1*	*APETALA 1*
*AtFUL*	*FRUITFULL*
*AtQQS*	*Arabidopsis Qua-Quine Starch*
*BR1*	*BOLTING RESISTANCE 1*
*BR2*	*BOLTING RESISTANCE 2*
*BR3*	*BOLTING RESISTANCE 3*
*BR4*	*BOLTING RESISTANCE 4*
*BrAGL9a*	*AGAMOUS-LIKE 9a*
*BraRGL1*	*RGA-LIKE1*
*BraXTH3*	*Xyloglucan endotransferase genes*
*BrOGs*	*Brassica rapa orphan genes*
*BrSDG8*	*SET DOMAIN GROUP 8*
*BrVIN3.1*	*VERNALISATION INSENTIVE 3.1*
*FD*	*FLOWER LOCUS D*
*FLC*	*FLOWERING LOCUS C*
*FRI*	*FRIGIDA*
*FT*	*FLOWER LOCUS T*
GA	Gibberellin
*LFY*	*LEAFY*
MS	Murashige and Skoog
OE	Overexpression
*OGs*	Orphan genes
*PpARDT*	*ABA-RESPONSIVE DROUGHT TOLERANCE*
qRT-PCR	Quantitative real-time polymerase chain reaction
*SOC1*	*SUPPRESSOR OF OVEREXPRESSION OF CONSTANS 1*
*TaFROG*	*FUSARIUM RESISTANCE ORPHAN GENE*
*TaSnRK1*	*SUCROSE NON-FERMENTING1-RELATED KINASE1*
Ver	Vernalization
