# Supplementary material for: Construction of an Overexpression Library for Chinese Cabbage Orphan Genes in Arabidopsis and Functional Analysis of BOLTING RESISTANCE 4-Mediated Flowering Delay
Source: Plants (Basel). 2025 Jun 25;14(13):1947. doi: 10.3390/plants14131947 (PMC12252378; doi:10.3390/plants14131947)
Supplement: Supplementary file 1 [file plants-14-01947-s001.zip › plants-3721884-Supplementary Figures.pdf]

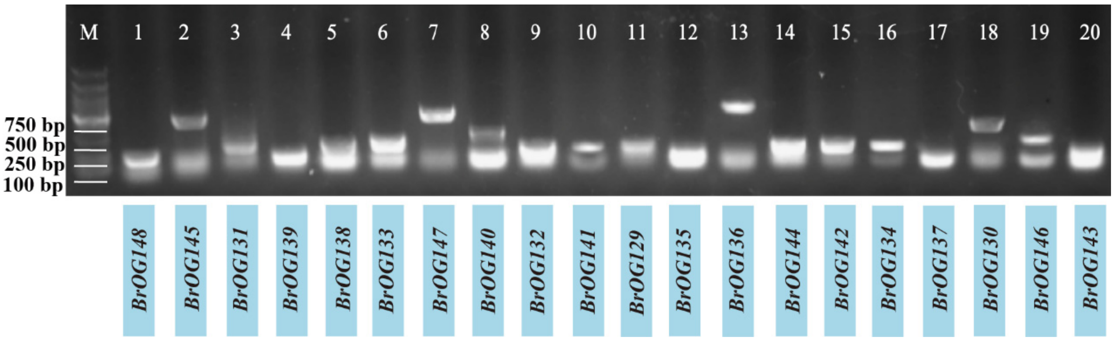

Supplementary Figure S1. PCR detection results of *BrOGs* cloning.

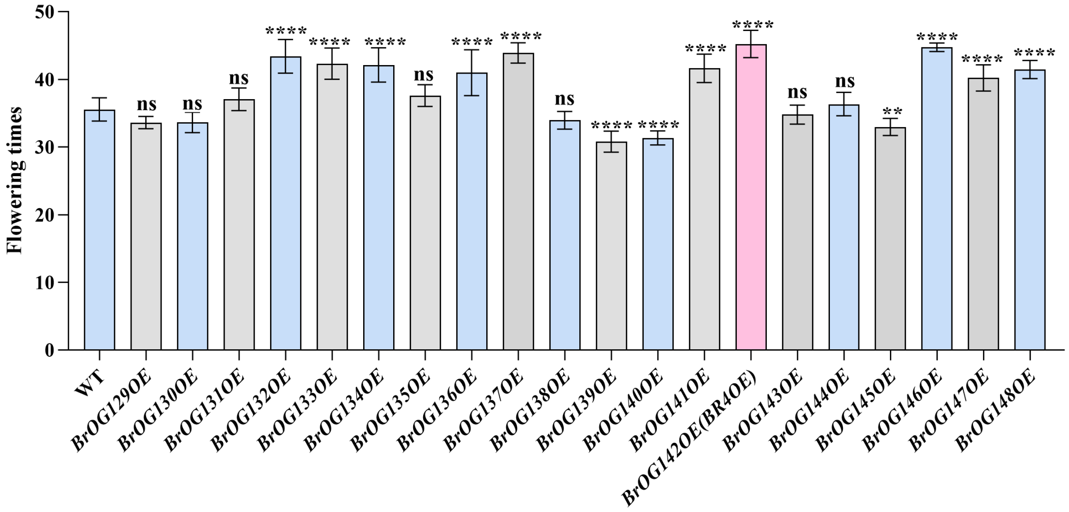

Supplementary Figure S2. Flowering time of *BrOGs* overexpression lines. All data are shown as mean ± SD of three biological replicates, ns indicates no significant difference, Asterisk indicate a significant difference from the WT, shown by Student's *t*-test, *$p < 0.05$, **$p < 0.01$, ***$p < 0.001$, and ****$p < 0.0001$.

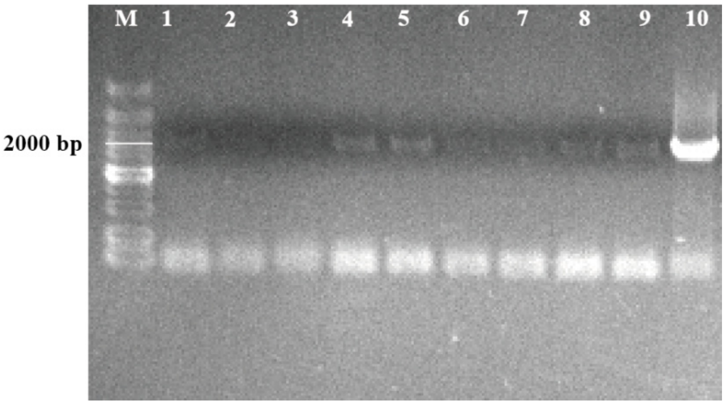

Supplementary Figure S3. Identification of GUS positive plants, M indicates 5000 marker, lane 10 was the positive control.
